# Temporal trends in the risk of developing multiple primary cancers: a systematic review

**DOI:** 10.1186/s12885-016-2876-y

**Published:** 2016-11-04

**Authors:** Yuanzi Ye, Amanda L. Neil, Karen E. Wills, Alison J. Venn

**Affiliations:** Menzies Institute for Medical Research, Univeristy of Tasmania, Private Bag 23, Hobart, Tasmania 7000 Australia

**Keywords:** Multiple Primary Cancers, Trends, Risk, Systematic review

## Abstract

**Background:**

Cancer survivors are at risk of developing second and subsequent primary cancers, referred to as multiple primary cancers (MPCs). It is not clear whether the risk of MPCs has increased over recent decades, but increasing use of radiological imaging and potentially harmful effects of certain cancer treatments raise this possibility. A systematic review was undertaken to assess whether there has been a temporal change in the risk of developing MPCs.

**Methods:**

A systematic search to identify population-based studies of MPCs was performed in Medline/PubMed and Embase databases from inception to August 2016. Included studies were those reporting risk of MPCs for all sites combined following a first cancer at any site or a specific site, using standard incidence ratios (SIRs) or equivalent, and with analysis stratified by calendar years.

**Results:**

We identified 28 articles eligible for inclusion, comprising 26 population-based studies and two monographs. MPC incidence was reported in nearly 6.5 million cancer survivors. For all first cancer sites combined, a higher rate of MPCs was reported in more recent than earlier calendar periods in four of the six relevant studies. The SIRs ranged from 1.14 for a first cancer diagnosis in the early 1980s to 1.21–1.46 in the late 1990s in the USA and Australia. Two studies from Italy and France showed no significant difference in SIRs across time periods 1978–2010 and 1989–2004. The remaining 22 studies reported various temporal trends in the risk of developing MPCs after a first cancer at a specific site, but most showed little change.

**Conclusion:**

Overall, the risk of developing MPCs appears to have increased since the 1980s when considering studies of all primary cancer sites combined from the USA and Australia but not from Europe. With the introduction of more routine nuclear medical imaging over the last 15 years, more studies are needed to confirm recent trends of MPC risk in adult cancer survivors.

**Electronic supplementary material:**

The online version of this article (doi:10.1186/s12885-016-2876-y) contains supplementary material, which is available to authorized users.

## Background

Survival for most cancers has increased steadily over the last three decades, mainly due to increased detection of early-stage cancers and advances in cancer treatment [1, 2]. This has been a global phenomenon and has led to a growing number of cancer survivors worldwide [3, 4]. Increasing attention has been given to the long-term outcomes of cancer survivors including the risk of developing new primary cancers [5, 6]. In a seminal report from the USA, up to 10 % of cancer survivors were diagnosed with a second or higher-order primary cancers during a 27-year period 1973 to 2000 [7]. A higher rate of new cancers was observed among cancer survivors with a first cancer diagnosed in more recent (between 1995 and 2000) than in earlier time periods (1973–79).

Two or more primary cancers occurring in the same individual that are neither extensions, recurrences nor metastases of each other are defined as Multiple Primary Cancers (MPCs) [8]. Factors associated with any change in the risk of developing MPCs might include increased use of diagnostic imaging and adverse cancer treatment effects. The past 30 years has seen a large increase in the use of diagnostic imaging, particularly radiologic medicine examinations such as diagnostic X-rays and computed tomography (CT) scanning [9–11]. Medical radiation exposure to the USA population has increased approximately 600 % since the 1980s [12]. In addition, cancer survivors tend to receive more frequent radiologic imaging than the general population due to follow-up care after primary treatment [13–15]. The rising use of various imaging modalities might be expected, therefore, to increase the possibility of incidental findings of new cancers during a routine follow-up examination and/or may increase the future risk of cancer due to the radiation exposure [16].

Some MPCs may also be treatment-related [17, 18]. Patients treated with radiotherapy and some specific chemotherapeutics can experience a number of significant late effects. One of the most serious potential long-term side effects is the development of MPCs [19–21]. The risk of developing MPCs is increased among survivors treated with radiotherapy, alkylating agents, anthracyclines and epipodophyllotoxins [3, 21–23]. A mutation in a susceptibility gene may also promote two or more cancers in an individual [22–24]. However, genetic risk factors for MPCs would not be expected to change over recent decades, unless they interact with other risk factors that demonstrate temporal trends.

In order to better understand temporal trends in the risk of developing MPCs, we performed a systematic review of the scientific literature to determine whether the risk of MPCs has increased over recent decades.

## Methods

### Scope of the review

We conducted a systematic literature search to identify studies describing adult cancer survivors with the diagnosis of MPCs. The review was focused on the following question: has there been an increase in the risk of developing MPCs over time?

### Search strategy and selection criteria

We used two approaches to conduct the systematic search in two phases (Table 1). The original review was conducted in PubMed and Embase databases for eligible articles published prior to 1st March 2015. The update was conducted to August 2016. The MeSH terms related to “multiple primary cancers” and “second cancers” and related subcategories were used in separate searches: Neoplasms/Multiple Primary, Neoplasms/Second Primary and epidemiology/prevention and control. A number of key words (“multiple primary cancer* or malignanc* or tumo*”, “population-based” and “time period* or interval* or calendar years”) were also used and combined in different databases.

**Table 1:** Search strategy for Medline and Embase (1 March 2015)

Approach 1	
Search strategy using MeSH terms in Medline	
No.	Search
1	“Neoplasms, Multiple Primary/epidemiology”[Mesh] OR "Neoplasms, Multiple Primary/prevention and control"[Mesh] OR "Neoplasms, Second Primary/epidemiology"[Mesh] OR "Neoplasms, Second Primary/prevention and control"[Mesh] AND "Time Factors"[Mesh]
	*N* = 657
2	Limits: adults
	*N* = 498
Search strategy using keywords in Medline	
1	multiple primary cancer*[Title/Abstract]
	*N* = 576
2	multiple primary malignanc*[Title/Abstract]
	*N* = 208
3	multiple primary tumo*[Title/Abstract]
	*N* = 386
4	multiple primary carcinoma*[Title/Abstract]
	*N* = 130
5	second cancer*[Title/Abstract]
	*N* = 1,272
6	second malignanc*[Title/Abstract]
	*N* = 1,622
7	second tumo*[Title/Abstract]
	*N* = 951
8	second carcinoma*[Title/Abstract]
	*N* = 82
9	#1 OR #2 OR #3 OR #4 OR #5 OR #6 OR #7 OR #8
	*N* = 4,801
10	time[Title/Abstract] OR period*[Title/Abstract] OR interval*[Title/Abstract] OR calendar year*[Title/Abstract]
	*N* = 3,401,312
11	population[Title/Abstract]
	*N* = 936,431
12	risk[Title/Abstract]
	*N* = 1,328,908
13	#9 AND #10 AND #11 AND #12
	*N* = 302
17	Limits: adult: 19+ years
	*N* = 236
Search strategy using keywords in Embase	
1	Multiple AND primary AND (‘cancer’/exp OR cancer) OR multiple AND primary AND malignanc* OR multiple AND primary AND tumor* OR multiple AND primary AND carcinoma* OR second AND (‘cancer’/exp OR cancer) OR second AND malignanc* OR second AND tumor* OR second carcinoma*
	*N* = 236,978
2	Time AND period* OR time AND interval* OR calendar AND year*
	*N* = 691,550
2	#1 AND #2 AND population AND risk AND [embase]/lim
	*N* = 457
Approach 2	
Search strategy using MeSH terms in Medline	
No.	Search
1	"Neoplasms, Multiple Primary"[Mesh] OR "Neoplasms, Multiple Primary"[Mesh] OR "Neoplasms, Second Primary"[Mesh] OR "Neoplasms, Second Primary"[Mesh] AND "Risk Assessment"[Mesh]
	*N* = 613
2	Limits: adults
	*N* = 455
Search strategy using keywords in Medline	
1	multiple primary cancer*[Title/Abstract]
2	multiple primary malignanc*[Title/Abstract]
3	multiple primary tumo*[Title/Abstract]
4	multiple primary carcinoma*[Title/Abstract]
5	multiple cancer*[Title/Abstract]
6	multiple malignanc*[Title/Abstract]
7	multiple tumo*[Title/Abstract]
8	multiple carcinoma*[Title/Abstract]
9	second primary cancer*[Title/Abstract]
10	second primary malignanc*[Title/Abstract]
11	second primary tumo*[Title/Abstract]
12	second primary carcinoma*[Title/Abstract]
13	second primary cancer*[Title/Abstract]
14	second primary malignanc*[Title/Abstract]
15	second primary tumo*[Title/Abstract]
16	second primary carcinoma*[Title/Abstract]
17	#1 OR #2 OR #3 OR #4 OR #5 OR #6 OR #7 OR #8 OR #9 OR #10 OR #11 OR #12 OR #13 OR #14 OR #15 OR #16
	*N* = 12,841
18	time OR period* OR year*
	*N* = 5,764,460
19	population-based[Title/Abstract]
20	risk[Title/Abstract]
21	#17 AND #18 AND #19 AND #20
	*N* = 232
17	Limits: adult: 19+ years
	*N* = 186

Following the Preferred Reporting items for Systematic reviews and Meta-analyses (PRISMA) statement [25, 26], eligibility criteria for included studies were as follows: (i) Type of studies: published population-based studies and reports published in English; (ii) Types of patients: adult cancer survivors (≥19 years) who were diagnosed with a first primary cancer (index cancer); (iii) Types of outcomes measures: adult cancer survivors (≥19 years) who developed a second or higher-order primary cancer (all sites combined). Studies of cancer survivors who developed MPCs at a specific site and studies based on autopsy cases were excluded because we were interested in the overall MPC risk among adult cancer survivors. Studies of MPCs in patients undergoing specific therapies or by treatment periods were also excluded given we were interested in all factors that affected the trends in MPC risk rather than treatment effects only.

### Data extraction and analysis

Titles and abstracts of identified articles were assessed against the inclusion criteria by one author (YY). The full text of potentially relevant studies and the reference lists of included studies were read to identify further original articles. Two authors (YY and AV) developed an extraction sheet to record first author’s name, publication year, source of data, the number of Strengthening the Reporting of Observational Studies in Epidemiology (STROBE) criteria met, site of first primary cancer, study period and follow-up, study size, study population (definition and inclusion criteria), definition of MPCs, calendar year of first cancer diagnosis, and the standardised incidence ratios (SIRs) or relative risks (RRs) and 95 % confidence intervals (95 % CIs) for MPCs by time periods. Typically, SIRs were derived from the observed number of MPCs divided by the expected number (O/E), with the expected number calculated from age-, sex- and calendar year- specific incidence rates in the general population [7, 27]. Alternatively, RRs were calculated as the risk of MPCs occurring in one time period compared with a reference period [28].

The Strengthening the Reporting of Observational Studies in Epidemiology (STROBE) criteria were used to assess the strengths, weaknesses, and generalizability of included studies [29]. The STROBE statement was developed to help readers when critically appraising published articles. Two authors (YY and AV) used a modified checklist of items for cohort studies to assess the number of criteria met in each study. We evaluated the coding rules of MPCs (i.e. Surveillance, Epidemiology, and End Results (SEER) [30] or International Association of Cancer Registries (IACR) and the International Agency for Research on Cancer (IARC) (IARC/IACR) [8] coding rules for MPCs) applied in each study as the diagnostic criteria in the STROBE checklist (Additional file 1).

## Results

### Literature search

**Fig. 1:**
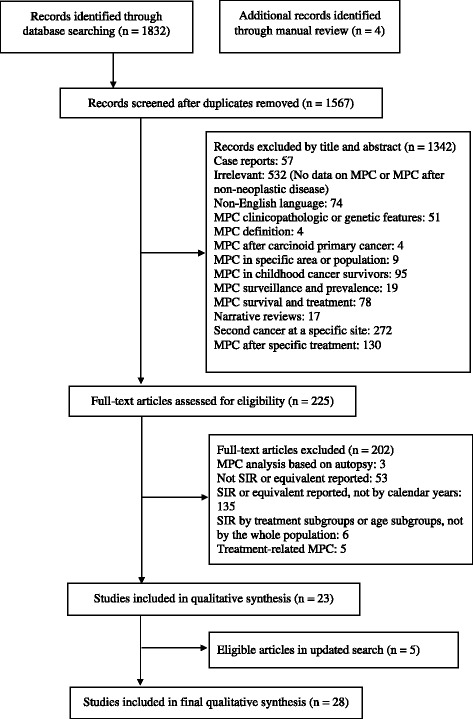
Flow diagram of study selection

The defined search criteria identified 1832 relevant articles and four were added through manual review of references. Of the 225 articles assessed as eligible for full-text review, 23 articles met the inclusion criteria and were included in the narrative synthesis. After combining five eligible articles in the updated search, 28 studies were included in the final analysis comprising 26 population-based studies and two monographs (Fig. 1).

### Study characteristics

All 28 included studies were population-based, published between 1987 and 2015, presenting data from Europe, North America, Australia and Japan (Table 2). 26 were peer-reviewed publications, reporting on more than 2.8 million survivors of adult cancer over the period of 1943 to 2012 [31–56], with 178,091 MPCs identified. Four of them reported the risk of developing MPCs following first cancer with all sites combined [45, 46, 48, 52]. Others focused on the risk of MPCs following first cancer at a specific site. The remaining were two monographs from the USA and Italy. One was a SEER monograph that used data from nine cancer registries in the USA, reported on more than 2 million cancer survivors during the follow-up period from 1973 to 2000, and a total of 185,407 MPCs were observed [7]. The other was a monograph of the Italian Association of Cancer Registries (AIRTUM), using data from 38 general and five specialised cancer registries in Italy, that reported on more than 1.6 million cancer survivors during the period of 1976–2010 with 85,399 MPCs identified [57].

**Table 2:** MPCs following a first primary cancer at any site or a specific site

First author, Publication year, Institute,	STROBE criteria (met/total criteria)	First cancer diagnosis	Design Study period Follow-up	Patients, N	Patients with MPCs, N	Study population: definition and inclusion criteria	Definition of MPCs: inclusion criteria	Calendar year of first cancer diagnosis	Standardised incidence ratio (95%CI)	Relative risk (95%CI)
Any site										
Curtis RE et al. (2006) [7] SEER, U.S.	27/30	Any site	1973-2000	More than 2 million	185,407	The study population includes nearly 2 million cancer patients reported to the 9 SEER registries from 1973 to 2000, with follow-up for subsequent cancer occurrence extending up to 27 years.	MPC coding rules: SEER	1973-1979	1.12	
								1980-1984	1.14	
								1985-1989	1.14	
								1990-1994	1.14	
								1995-2000	1.21	
AIRTUM Working Group (2013) [57] Italian Association of Cancer Registries, Italy	27/30	Any site	1976-2010	1,635,060	85,399	This monograph uses data from the AIRTUM Database (at December 2012) regarding all cancer cases, except non-melanoma skin cancer, diagnosed between 1976 and 2010 in the general cancer registries.	MPC coding rules: IARC/IACR rules	1978-1987	1.10 (1.09-1.11)	
								1988-1997	1.08 (1.07-1.10)	
								1998-2010	1.10 (1.09-1.12)	
Jégu J et al. (2014) [48] 10 French population-based cancer registries, France	28/30	Any site	1989-2004 Followed up to 2007	289,967	21,226	All patients presenting with a first cancer diagnosed between 1989 and 2004, excluding non-melanoma skin cancers.	MPC coding rules: IARC/IACR rules	1989-1994	1.39 (1.36-1.42)	
								1995-1999	1.36 (1.33-1.39)	
								2000-2004	1.34 (1.30-1.37)	
Youlden DR et al. (2011) [46] Queensland Cancer Registry, Australia	26/30	Any site	1982-2001 Followed up to 2006	204,962	23,580	All patients diagnosed with a first primary invasive cancer between 1982 and 2001 who survived for a minimum of 2 months, restricting to 15 years or older at the time of first diagnosis.	MPC coding rules: Included histologically similar cases of cancer at the same body site. Excluded synchronous primary cancers (those diagnosed within 2 months of the first primary cancer)	1982-1986	1.14 (1.08-1.20)	
								1987-1991	1.22 (1.17-1.28)	
								1992-1996	1.36 (1.31-1.41)	
								1997-2001	1.46 (1.41-1.50)	
Sankila R et al. (1995) [52] Finnish Cancer Registry, Finland	22/30	Any site	1953-1991	470,000	19,800	All 470,000 patients registered in Finland from 1953 to 1991 with malignant neoplasms [primary site codes 140–208 in the International Classification of Diseases (ICD-7), WHO, 19571 excluding basal-cell carcinomas of the skin, carcinoma in situ of the uterine cervix, and papilloma of the urinary organs.	MPC coding rules: IARC/IACR rules	1953-1959	1.09 (1.05-1.13)	
								1960-1969	1.11 (1.08-1.14)	
								1970-1979	1.11 (1.08-1.13)	
								1980-1991	1.14 (1.11-1.16)	
Tsukuma H et al. (1994) [45] Osaka Cancer Registry, Japan	18/30	Any site	1966-1986 Followed up to 1989	217,307	4,436	All reported cases aged 0–79 who were initially diagnosed with a first primary cancer (invasive cancer and benign intracranial tumour)	MPC coding rules: IARC/IACR rules		Ratio of SIRs	
								1966-1971	1.00 (reference)	
								1972-1977	1.59 (1.33-1.90)	
								1978-1983	2.89 (2.47-3.38)	
								1984-1986	2.89 (2.45-3.40)	
Specific site										
leukaemia, lymphoma and myeloma										
Rebora P et al. (2010) [35] Swedish Cancer Registry, Sweden	23/30	Chronic myeloid leukaemia	1970-1995 Followed up to 2007	2,753	145	All adult cases of CML as a primary diagnosis (ICD-7 code 205.1, age at diagnosis ≥ 18 years) arising between January 1, 1970, and December 31, 1995.	MPC coding rules: not specified.	1970-1984	1.68 (1.32-2.12)	
								1985-1995	1.97 (1.55-2.48)	
Schöllkopf C et al. (2007) [36] Danish Cancer Register, Denmark	24/30	Chronic lymphocytic leukaemia	1943-2003	12,373	1,105	All patients with chronic lymphocytic leukaemia (ICD-7-code 204.0)	MPC coding rules: not specified.	1943- 1994	1.62 (1.50-1.76)	
							Excluding second cancers diagnosed less than one year after CLL	1994- 2003	1.55 (1.41-1.69)	
Hisada M et al. (2007) [40] SEER, U.S.	26/30	Hairy cell leukaemia	1973-2002	3104	358	All hairy cell leukaemia patients who survived for at least 2 months after diagnosis.	MPC coding rules: SEER	1973-1989	1.17 (1.01-1.36)	
								1990-2001	1.30 (1.12-1.51)	
Federico M et al. (2002) [51] The nationwide registry of the Italian Cooperative Group, Italy	22/30	Hairy cell leukaemia	1978-1999	952	49	Patients were recorded in the Italian Registry of HCL between January 1981 and December 1996.	MPC coding rules: not specified.	1978-1982	1.00 (0.27-2.57)	
								1983-1987	0.89 (0.36-1.84)	
								1988-1992	1.04 (0.62-1.65)	
								1993-1999	1.02 (0.62-1.58)	
M.P. Coleman et al. (1987) [32] South Thames (now Thames) Cancer Registry, UK	28/30	Hodgkin’s disease	1961-1980 Followed up to 1981	2,970	58	All patients registered with Hodgkin’s disease in the South Thames Cancer Registry during the period 1961–80. Excluded patients if they had had another tumour registered either before or at the same time as the index tumour, or if their index tumour had been registered at death (no follow-up) or at age 85 years or more.	Second cancer defined as the site and the histology are distinct from the first. Second cancers at the same site as the first or at a different site but with the same histology as the first will be registered only if the hospital record or pathology report explicitly states that it is a new primary, distinct from the previous cancer. Excluded second tumours occurring at age 85 or over.	1961-1969	1.2	
								1970-1980	1.6	
Lorenzo Bermejo J et al. (2014) [34] Cancer registries of Finland, Norway and Sweden	22/30	non-Hodgkin lymphoma	1980-2006	21,036 Finnish,	6815	Almost all histologically confirmed cases of non-Hodgkin lymphoma.	MPC coding rules: not specified	1980-84		0.65 (0.59-0.72)
								1985-89		0.71 (0.66-0.77)
				14,027 Norwegian				1990-94		0.77 (0.72-0.83)
								1995-99		0.77 (0.73-0.82)
				25,838 Swedish				2000-06		Reference
Rossi C et al. (2015) [54] 10 French population-based cancer registries, France	25/30	non-Hodgkin lymphoma	1989-2004 Followed up to 2007	7,546	580	NHL patients was extracted from the K2 France cohort, which includes cancer cases diagnosed between 1989 and 2004 recorded by 10 French populationbased cancer registries. Patients who developed a synchronous second cancer (<61 days of follow-up) were excluded.	MPC coding rules: A Second Primry Cancer was defined as the first subsequent primary cancer occurring at least two months (≥61 days) after the first diagnosis of NHL.		Ratio of SIRs	
								1989-1994	1.00 (reference)	
								1995-1999	1.07 (0.86-1.34)	
								2000-2004	1.37 (1.08-1.74)	
Razavi P et al. (2013) [43] SEER, U.S.	28/30	Multiple myeloma	1973-2008	36,491	2021	All cases were identified by site code ICD-0-3: C9732 and C9734. Excluded cases whose reporting sources were coded as autopsy or death-certificate-only (*n* = 775), cases where MM was not the first primary (*n* = 3545) and cases with second cancer diagnosed within the first 2 months of MM diagnosis (*n* = 365)	MPC coding rules: SEER	1973-1984	1.04 (0.95-1.13)	
								1985-1999	0.95 (0.90-1.02)	
							All second cancers except cancers within the first two months after diagnosis of MM	2000-2008	0.96 (0.88-1.06)	
Breast and ovarian cancer										
Mellemkjaer L et al. (2006) [37] 13 population-based cancer registries Europe, Australia, Canada and Singapore	26/30	Breast cancer	1943-2000	525,527	31,399	All women with a first primary breast cancer (ICD-9 5 174) except patients for whom the first primary cancer diagnosis and death were recorded at the same time or who had 2 first primary cancers recorded simultaneously (same dates of diagnosis).	MPC coding rules: IARC/IACR	<1975	1.32 (1.30-1.35)	
								1975-1983	1.22 (1.20-1.25)	
							Second cancers except contralateral breast cancer, brain and nervous system only included malignant tumours, bladder cancer included papilloma, included all non-melanoma skin cancer	1984-1990	1.23 (1.20-1.26)	
								1991+	1.18 (1.14-1.22)	
Brown LM et al. (2007) [31] Population-based cancer registries in Denmark, Finland, Norway and Sweden	27/30	Breast cancer	1943-2002	376,825	23,158	All women diagnosed with a first primary cancer of the breast between January 1, 1943 and December 31, 2002 who survived at least 1 year.	MPC coding rules: Subsequent primary non-haematological malignancies (except breast cancer) that developed at least 1 year after breast cancer diagnosis	<1980	1.19^a^	
								≥1980	1.09^a^	
									^a^ Confidence interval does not include 1.0	
Molina-Montes E et al. (2013) [38] Granada Cancer Registry, Spain	26/30	Breast cancer	1985-2007	5897	314	All cases were identified by site code ICD-O-3 (C50).	MPC coding rules: IACR/IARC	1985-1995	1.37 (1.16-1.58)	
						Exclude patients whose first primary cancer diagnosis and death were recorded simultaneously and synchronous first primary cancers.	All second primary cancers except contralateral breast cancer which considered as a single tumour.	1996-2007	1.41 (1.18-1.64)	
M.P. Coleman et al. (1987) [32] South Thames (now Thames) Cancer Registry, UK	28/30	Ovarian cancer	1961-1980 Followed up to 1981	11,802	170	All patients registered with cancer of the ovary in the South Thames Cancer Registry during the period 1961–80. Excluded patients if they had had another tumour registered either before or at the same time as the index tumour, or if their index tumour had been registered at death (no follow-up) or at age 85 years or more.	Second cancer defined as the site and the histology are distinct from the first. Second cancers at the same site as the first or at a different site but with the same histology as the first will be registered only if the hospital record or pathology report explicitly states that it is a new primary, distinct from the previous cancer. Excluded second tumours occurring at age 85 or over.	1961-1969	1.1	
								1970-1980	1.2	
Thyroid cancer										
Cho YY et al. (2015) [49] Korean Central Cancer Registry, Korea	25/30	Thyroid cancer	1993-2010	178,844	2,895	Records code C73.9 starting in January 1993 through December 2010. Patients who developed a second malignancy within the first 2 months of follow-up (*n* = 628) were excluded.	MPC coding rules: not specified.	1993-1997	0.98 (0.90-1.07)	
								1998-2002	1.05 (0.98-1.13)	
								2003-2007	1.09 (1.03-1.15)	
								2008-2010	1.06 (0.96-1.17)	
Kim C et al. (2013) [42] SEER, U.S.	24/30	Thyroid cancer	1973-2008	52,103	4457	All cases were identified by site code ICD-0-3: C739, reported to a SEER 9 database between 1973-2008	MPC coding rules: SEER All second cancers except cancers within the first two months after initial thyroid cancer	1973-1983	1.02 (0.97-1.07)	
								1984-1993	1.03 (0.97-1.08)	
								1994-2003	1.21 (1.14-1.28)	
								2004-2008	1.45 (1.28-1.62)	
Prostate cancer										
Joung JY et al. (2015) [55] Korean Central Cancer Registry, Korea	22/30	Prostate cancer	1993-2011	55,378	2,578	Patients diagnosed with a first prostate cancer between 1993 and 2011. Excluded patients who presented with a SPC within two months of their first prostate cancer diagnosis, patients with subsequent prostate cancer after the diagnosis of another primary cancer, and patients for whom only death certificate information was available.	MPC coding rules: not specified	1993-2000	0.6	
								2001-2011	0.7	
Levi F et al. (1999) [33] Vaud and Neuchâtel Cancer Registry, Switzerland	26/30	Prostate carcinoma	1974-1994	4,503	380	Cases of first diagnosed prostate carcinoma registered between 1974 and 1994 with histologic confirmation available for 89.7 %.	MPC coding rules: not specified	1974-1984	0.7 (0.6-0.8)	
								1985-1994	0.7 (0.6-0.8)	
Other sites (malignant meningioma, head and neck, oesophageal, ocular melanoma, merkel cell, colorectal cancer, bladder, testis)										
Bao X et al. (2014) [39] SEER, U.S.	26/30	Malignant meningioma	1973-2007	1,603	56	All patients in the SEER database with the diagnosis of malignant meningioma were identified via SEER program 6.6.2 (1973–2007).	MPC coding rules: SEER	1973-1988	0.78	
								1989-1999	1.01	
								2000-2007	0.56	
Jégu J et al. (2013) [50] Bas-Rhin populationbased cancer registry, France	28/30	Head and neck squamous cell carcinomas	1975-2006	7,329	1,326	All patients were followed-up for 10 years or until December 31, 2006. HNSCC included here were squamouscell carcinomas (ICD-O-3 histology codes 8070–8076, 8078) localized at the oral cavity, oropharynx, hypopharynx and larynx (ICD-O-3 site codes C01–C06, C09–C10, C12–C13, C32).	MPC coding rules: IARC/IACR rules		Ratio of SIRs	
								1975-1979	1.00 (reference)	
								1980-1984	1.15 (0.95-1.40)	
								1985-1989	1.29 (1.06-1.55)	
								1990-1994	1.25 (1.03-1.51)	
								1995-1999	1.10 (0.90-1.35)	
								2000-2006	0.85 (0.67-1.08)	
Zhu G et al. (2012) [44] SEER, U.S.	25/30	Oesophageal cancer	1973-2007	24,557	985	All oesophageal cancer patients who survived for at least 2 months after diagnosis.	MPC coding rules: SEER All second primary cancers except non-melanoma skin cancers	1973-1989	1.43 (1.29-1.58)	
								1990-2007	1.28 (1.18-1.38)	
Scélo G et al. (2007) [47] 13 population-based cancer registries Europe, Australia, Canada and Singapore	25/30	Ocular melanoma	1943-2000	10,396	1,029	All cases of ocular melanoma without stratifying by subsite.	MPC coding rules: IARC/IACR rules	<1975	1.17 (1.06-1.29)	
								1975-1983	1.21 (1.08-1.35)	
								1984-1990	1.39 (1.20-1.59)	
								1991+	1.33 (1.08-1.62)	
Howard RA et al. (2006) [41] SEER, U.S.	26/30	Merkel cell carcinoma	1986-2002	1,306	122	All patients with a first primary cutaneous MCC in 1 of 11 population-based cancer registries of SEER program (1986–2002).	MPC coding rules: SEER Subsequent primary cancers were invasive primary neoplasms that developed at least 1 month after a diagnosis of MCC. Excluded secondary MCC following primary MCC.	1986-1994	1.09 (0.83-1.40)	
								1995-2002	1.37 (1.05-1.76)	
Guan X et al. (2015) [53] SEER, U.S.	23/30	Colorectal cancer	1992-2012	240,584	27,731	Invasive CRC patients who were diagnosed	MPC coding rules: SEER	Colon		
								1992-2001	1.08	
						at the age of more than 20 years. Excluded patients: 1) diagnosed with unknown age, 2) reported only on death or autopsy certificate only, 3) being stage of in situ. SPMs diagnosed during six months period after the primary diagnosis were also excluded.		2002-2012	1.25	
								Rectum		
								1992-2001	1.00	
								2002-2012	1.16	
Muller J et al. (2015) [56] 10 French population-based cancer registries, France	28/30	Bladder cancer	1989-2004 Followed up to 2007	10,047	1,291	All patients with a first bladder cancer (BCa) diagnosed between 1989 and 2004 and followed up to 31 December 2007. Excluded patients with a known history of previous cancer before BCa diagnosis	MPC coding rules: IARC/IACR rules	1989-1992	1.53 (1.37-1.71)	
								1993-1996	1.42 (1.27-1.58)	
								1997-2000	1.57 (1.41-1.74)	
								2001-2004	2.02 (1.79-2.27)	
M.P. Coleman et al. (1987) [32] South Thames (now Thames) Cancer Registry, UK	28/30	Testicular cancer	1961-1980 Followed up to 1981	2,013	27	All patients registered with cancer of the testis in the South Thames Cancer Registry during the period 1961–80. Excluded patients if they had had another tumour registered either before or at the same time as the index tumour, or if their index tumour had been registered at death (no follow-up) or at age 85 years or more.	Second cancer defined as the site and the histology are distinct from the first. Second cancers at the same site as the first or at a different site but with the same histology as the first will be registered only if the hospital record or pathology report explicitly states that it is a new primary, distinct from the previous cancer. Excluded second tumours occurring at age 85 or over.	1961-1969	0.8	
								1970-1980	0.7	

*STROBE* Strengthening the Reporting of Observational Studies in Epidemiology, *MPCs* multiple primary cancers, *N* number of patients, *SEER* Surveillance, Epidemiology, and End Results, *IARC/IACR* International Association of Cancer Registries (IACR)/the International Agency for Research on Cancer (IARC), *CI* confidence interval, *ICD* International Classification of Diseases, *CML* chronic myeloid leukaemia, *CLL* chronic lymphocytic leukaemia, *MM* multiple myeloma, *MCC* Merkel cell carcinoma

The coding rules to define MPCs varied across studies. Seven studies and the SEER monograph used incidence and follow-up data from SEER program registries, and employed SEER coding rules [7, 39–44, 53]. Eight studies and the Italian monograph used coding rules proposed by the IARC/IACR [37, 38, 45, 47, 48, 50, 52, 56, 57]. While it may be difficult to directly compare risk estimates derived using different coding rules, comparisons of temporal trends will be valid if the rules used to define MPCs are consistent over time within a single study population [58].

### Study quality

The number of STROBE criteria met in all population-based studies ranged from 18 to 28 of 30 items in total, with 21 studies meeting at least 25 criteria. Included studies had various objectives, data sources, study sizes and STROBE criteria met, but they all reported the risk of MPCs over different time periods.

### Risk of MPCs following first primary cancer with all sites combined

Six studies reported temporal trends in MPC risk among survivors of adult cancer with all first cancer sites combined. Four of them observed an increasing trend in MPC risk from earlier to more recent periods. The SEER monograph reported a 14 % higher risk of developing MPCs than would be expected in the general SEER population during the 25 years of follow-up, with a total of 185,407 observed MPCs compared with 162,602 expected (SIR, 1.14; 95 % CI, 1.14 to 1.15). There was an increasing trend of SIRs rising from 1.12 with periods of first cancer diagnosis during 1973–79 to 1.21 during 1995–2000 [7]. Three large population-based studies from Australia, Finland and Japan (Australia and Japan including more than 200,000 cancer survivors, Finland including 470,000 cancer survivors) also showed an increase in the risk of developing MPCs across the whole study period when all first cancer sites are combined. In Australia, the SIRs grew from 1.14 with periods of first diagnosis in 1982–1986 to 1.46 in 1997–2001 [46]. In Finland, the SIRs increased from 1.09 in the 1950s to 1.14 in the 1980s [52]. In Japan, the relative risk increased from 1.00 in 1966–1971 (reference) to 2.89 in 1984–1986 [45]. However, two studies from Italy and France showed no significant difference in SIRs across different time periods. The Italian monograph reported SIRs of 1.10 in 1978–1987, 1.08 in 1988–1997 and 1.10 in 1998–2010, with a 10 % higher risk of developing MPCs than expected across the entire study period 1976–2010 (SIR, 1.10; 95%CI, 1.09 to 1.10) [57]. The French study was a large population-based study using data from 10 French population-based cancer registries, with a first cancer diagnosis between 1989 and 2004 [48].

### Risk of MPCs following first primary cancers at specific sites

The risk of MPCs following first cancers at specific sites did not differ significantly across calendar periods of first cancer diagnosis in 14 studies [33, 35, 36, 38–41, 43, 44, 47, 49–51, 55]. Six studies reported an increasing temporal trend in MPC risk [32, 34, 42, 53, 54, 56], whilst two studies reported a decreasing trend in MPC risk after breast cancer during the study period 1943–2000 [31, 37]. There were a total of three studies on breast cancer as the first cancer.

#### MPCs following leukaemia, lymphoma and myeloma

Eight studies assessed the risk of MPCs following first cancers of the hematopoietic and lymphoid system [32, 34–36, 40, 43, 51, 54]. The risk of MPCs did not reveal any particular trends with respect to variations in SIRs over time in either of two major leukaemia (chronic myeloid leukaemia, chronic lymphocytic leukaemia) [35, 36] or the uncommon hairy cell leukaemia [40, 51]. These studies compared the risk of MPCs before and after a time point around 1990 when novel therapeutics such as interferon, fludarabine and other nucleoside analogues were introduced. Three studies with first cancers of Hodgkin or non-Hodgkin lymphoma reported an increasing trend in the risk of MPCs over time [32, 34, 54]. The risk of MPCs increased from 1.2 in the 1960s to 1.6 in the 1970s among 3139 cases of Hodgkin’s disease [32]. For MPCs following non-Hodgkin lymphoma (NHL), the risk was higher in the time interval of 2000–2006 (RR = 1.00, reference) than in 1980–1984 (RR = 0.65; 95%CI: 0.59-0.72) in more than 60,000 registered cases from three Nordic countries [34]. A similar pattern was also confirmed in France. The risk of MPCs was 1.37 (95%CI: 1.08-1.74) times higher for NHL diagnoses in 2000–2004 than in the 1989–1994 reference category [54]. For MPCs following multiple myeloma, there was no significant change in the risk before and after 2000. However, the overall risk of MPCs was also not significant (SIR = 0.98; 95 % CI: 0.94–1.02) [43].

#### MPCs following breast and ovarian cancer

No statistically significant change or decreasing trends in MPCs risk was observed in three breast cancer studies, two from multicentre studies and one from a single cancer registry [31, 37, 38]. One large multicentre study used data on 525,527 breast cancer survivors from 13 population-based cancer registries from Europe, Australia, Canada and Singapore covering the study period 1943–2000. The risk of MPCs decreased from 1.32 (95%CI 1.30-1.35) before 1975 to 1.18 (95%CI 1.14-1.22) after 1991 [37]. Another multicentre study encompassing four Scandinavian cancer registries reported data on more than 300,000 one-year survivors of breast cancer during a similar period, 1943–2002. The risk of MPCs was lower after 1980 than before (SIR = 1.09 and 1.19, respectively). However, second haematological cancers were excluded from the analysis [31]. No significant change in trend was observed in a study from a single cancer registry in Spain during the period of 1985–2007 (SIR 1.37, 95%CI 1.16-1.58 in 1985–1995 and SIR 1.41, 95%CI 1.18-1.64 in 1996–2007) [38]. The risk of MPCs following ovarian cancer increased from 1.1 in 1961–69 to 1.2 in 1970–80 (no 95%CI provided), using data from a single cancer registry in the UK. However, the observed numbers were mostly too small to obtain reliable estimates [32].

#### MPCs following thyroid cancer

In the USA, the risk of MPCs was higher in patients diagnosed with a first thyroid cancer during 2004–2008 (SIR 1.45, 95%CI 1.28-1.62) than in 1973–1983 (SIR 1.02, 95%CI 0.97-1.07) [42]. In Korea, however, the risk only reached statistical significance for a first thyroid cancer diagnosis between 2003 and 2007 (SIR 1.09, 95%CI 1.03-1.15), with smaller increases in other periods (SIR = 0.92 in 1993–1997, 1.05 in 1998–2002 and 1.06 in 2008–2010) [49].

#### MPCs following prostate cancer

Interestingly, the risk of MPCs following a first prostate cancer was significantly lower than expected in two relevant studies from the USA and Korea [33, 55]. The value of SIRs was consistently 0.7 from 1974 to 1994 using data from SEER (USA) cancer registries [33] and ranged from 0.6 to 0.7 during 1993–2011 using data from a nationwide hospital-based cancer registry in Korea [55].

#### MPCs following first cancers at other sites

From the early 1970s to the mid-2000s, the temporal trends in the risk of MPCs did not change significantly with a first cancer diagnosis of malignant meningioma, oesophageal cancer and ocular melanoma [39, 44, 47], but varied for first cancer at head and neck [50]. The risk of MPCs following testicular cancer remained consistent and lower than expected from 1961 to 1980. Although the SIRs for first cancer of Merkel Cell carcinoma increased from 1.09 (95%CI 0.83-1.70) in 1986–1994 to 1.37 (95%CI 1.05-1.76) in 1995–2002, the increase was not statistically significant [41]. For MPCs following colorectal cancer and bladder cancer, both risks increased with the year of first cancer diagnosis. The SIRs increased significantly from 1.53 (95%CI 1.37-1.71) in 1989–1992 to 2.02 (95%CI 1.79-2.27) in 2001–2004 for first bladder cancer [56]. For first cancer at colon and rectum, the SIRs were higher in 2002–2012 (1.25 for colon, 1.16 for rectum) than in 1992–2001 (1.08 for colon, 1.00 for rectum) [53].

## Discussion

To our knowledge, this is the first systematic review focusing on the temporal trends in the risk of MPCs. There was an increasing time trend in the risk of developing MPCs in the USA and Australia when all first cancer sites were combined. Risk increased from 1.12 to 1.14 following a first cancer diagnosis in the early 1980s, to 1.21-1.46 in the late 1990s. In European countries, the risk remained similar during 1978–2010 for Italian cancer survivors and showed no significant change between 1989 and 2004 for French cancer survivors. Three potential explanations are postulated for the increasing trends in the USA and Australia: 1) increased detection of MPCs, both intended and incidental; 2) increased radiation exposure and 3) changed cancer treatments. The trends in the risk of developing MPCs varied by site of first cancer from 1943 to 2012, but mostly there was little change.

### Increased detection

Increasing risk of MPCs might be a result of increased detection arising from the introduction of cancer screening programs for the early detection of cancers in the community; and incidental findings arising from the increased use of sensitive imaging tests in the routine clinical follow-up of cancer survivors. Early detection and incidental findings are not necessarily of benefit, as they can lead to “overdiagnosis”. Overdiagnosis is the detection of a “cancer” that would not cause symptoms or death in a patient’s lifetime [59].

Higher MPCs risk was observed in studies from the USA and Australia in the late 1990s [7, 46]. These findings coincide with the introduction of national cancer screening programs for cervical cancer and breast cancer in Australia in the early 1990s [60]. In the USA, the use of cancer screening for cervical cancer, breast cancer, colorectal cancer and prostate cancer has increased since 1987, particularly for breast cancer [61–63]. As well as providing benefits, screening has led to the overdiagnosis of non-progressive or low progressing cancers [64–67].

Cancer survivors are considered at increased risk for future cancers, and screening has been specifically recommended for survivors of some cancer types [68]. A meta-analysis of 20 studies demonstrated that cancer survivors tended to receive more frequent screening for new primary cancers, especially for breast, cervical, colorectal and prostate cancer than non-cancer controls [69]. This may thus differentially lead to increased detection among cancer survivors compared with the general population.

Another activity leading to a higher rate of MPCs may be the increased use and improvements in diagnostic imaging in recent time periods. The use of diagnostic imaging, especially computed tomography (CT) scans, has increased dramatically worldwide since 1980, particularly in the USA, Australia and Japan [9, 11, 70]. Cancer survivors routinely undergo CT scanning and other imaging procedures during follow-up, the detection of a new primary cancer, therefore, becomes more likely than in the general population with no prior cancer diagnosis [71]. Some of these incidental findings might be clinically unimportant, leading to overdiagnosis [10, 59, 72].

### Increased radiation exposure during the follow-up after a first cancer diagnosis

Radiation exposure due to monitoring may of itself lead to harmful effects [73]. This possibility is consistent with our finding that the highest risk of MPCs, from the late 1990s, has occurred since radiation exposure has increased enormously [9]. Since the 1980s, the average annual per-capita effective dose from medical radiological procedures doubled worldwide [9, 11]. In the USA, the average annual per capita dose for medical procedures increased almost six-fold from 0.5 mSv in 1980 to 3.0 mSv in 2006 [9], with X-rays and CT scan the two most common imaging procedures leading to radiation exposure [11].

The lifetime risk of cancer attributable to diagnostic X-rays is estimated to vary between 0.6 and 1.8 % across 15 countries, including the USA and Australia, between 1991 and 1996 [74]. In Japan, the attributable risk has been estimated at 3.2 %, but the Japanese results are confounded by the impact of background radiation following the atomic bombings in World War II [74].

CT scanning has been widely used since the 1990s and delivers a much higher radiation dose than diagnostic X-rays [75]. In Australia, the cancer risk in children and adolescents who underwent CT scanning between 1985 and 2005 was found to be 24 % higher than in their peers who did not undergo CT scanning [16]. The impact on lifetime risk could not be ascertained given that cancer excess was still occurring at the end of the study. Increased risk of cancer due to CT scanning is not, however, limited to children. Adults are also at increased risk of cancer from the radiation exposure [76]. Imaging-based evaluation, especially CT scans, is the preferred modality to assess response to treatment, and for routine surveillance of most cancer survivors [77]. Therefore, increased risk of MPCs could be partly attributable to cumulative radiation exposure due to recurrent CT scans [78].

### Changed cancer treatments

Studies of MPCs consequent to a primary cancer at a specific site can help us understand the impact of treatment changes on trends of MPC risk. No statistically significant change in risk of MPC was observed in most specific site studies, including after the 1990s when many newer (and improved) treatments or management strategies were introduced [33, 35, 36, 38–41, 43, 44, 51, 55]. A decrease in risk was observed in two of three breast cancer studies, which may reflect the improvement in radiation techniques [31, 37]. On the other hand, an increase in MPC risk was observed for a first non-Hodgkin lymphoma diagnosis in the mid-2000s than in the early 1990s, which may suggest an adverse treatment effect with increased use of nucleoside analogues (e.g. Fludarabine) at the end of the 1990s [34, 54]. The increase in the risk of MPCs following thyroid cancer might occur as a consequence of aggressive radiation treatment [42]. This is particularly concerning in regard to the more recent increased detection and treatment of microcarcinoma [79], largely considered to encompass overdiagnosis and overtreatment [72].

Together these findings suggest that most treatments have not impacted the baseline risk of MPCs in persons recently diagnosed with a primary cancer (post-1990), with the exceptions of breast cancer (reduction), non-Hodgkin lymphoma and thyroid cancer (increase).

### Other factors to be considered

Changing patterns in lifestyle or environmental risk factors may also affect the value of SIRs over time. Temporal trends in population exposure to carcinogenic factors such as alcohol and tobacco consumption, differed across countries [48]. Any differences over time in exposure in the reference population compared with the population of cancer survivors could contribute to changing SIRs.

### Limitations

Several factors limit the interpretation of our findings. First, studies of all first cancer sites combined from the USA and Australia did not cover the last 15 years. This period is of particular relevance given the introduction of nuclear medicine imaging, for example, PET/CT scanning. Second, cancer survivor profile (age at first cancer diagnosis, site of first cancer) may have varied according to year of first cancer diagnosis and these factors may contribute to changing SIRs [48]. For example, cancer survivor profiles have changed with the implementation of cancer screening programs. Third, variation in screening guidelines, follow-up care, management and treatment for cancer across countries potentially affect the generalizability of the results. Fourth, some included studies did not clearly specify the coding rules or definition of MPCs, which may result in misclassification and less reliable estimation of the observed number of MPCs. Fifth, different timeframes of the study cohorts may limit the ability to evaluate long-term effects of changing patterns in medical surveillance and treatment of the primary cancer. Some studies published before 2000 lack sufficient data to compare SIRs pre-1990s and post-1990s when treatment improved [32, 33, 45, 52]. Sixth, articles in languages other than English were excluded, which could lead to language bias. Last, although we defined the age of the study population as adults, some studies, mostly those using SEER cancer registries, reported across all ages [7, 32, 34, 36, 39–45, 47, 49, 52, 54, 57]. However, childhood cancer survivors accounted for a limited proportion of all cancer survivors (no more than 2 % in SEER cancer registries), and the types of cancer developed in children were limited [7], which should minimise any impact on the overall results.

## Conclusion

The overall risk of developing MPCs appears to have increased in the USA and Australia from the 1980s to 2000 when all first cancer sites are combined. Increased detection due to the more frequent use of diagnostic imaging and increased medical radiation exposure may be potential explanations. Studies from Italy and France, however, showed no significant change in MPC risk in patients diagnosed with a first cancer after 2000. Given the implementation of new cancer screening programs and the growth of nuclear medical imaging (e.g. PET/CT scanning) since 2000, continued long-term surveillance is needed from non-European as well as European countries. Future studies are needed to assess the extent of overdiagnosis and overtreatment among cancer survivors relative to the general population, and thus to assess whether there is potential for optimising follow-up strategies.

## Additional file

Additional file 1: STROBE Statement—Checklist of items that should be included in reports of *cohort studies.* (DOC 87 kb)

## Abbreviations

CI: Confidence Interval
IARC/IACR: International Agency for Research on Cancer/International Association of Cancer Registries
MPCs: Multiple Primary Cancers
PRISMA: Preferred Reporting Items for Systematic Reviews and Meta-analyses
RR: Relative Risk
SEER: Surveillance, Epidemiology, and End Results
SIR: Standardised Incidence Ratio
STROBE: Strengthening the Reporting of Observational Studies in Epidemiology
